# Occurence of ArmA and RmtB Aminoglycoside Resistance 16S rRNA Methylases in Extended-Spectrum β-Lactamases Producing *Escherichia coli* in Algerian Hospitals

**DOI:** 10.3389/fmicb.2016.01409

**Published:** 2016-09-12

**Authors:** Amel Ayad, Mourad Drissi, Claire de Curraize, Chloé Dupont, Alain Hartmann, Sébastien Solanas, Eliane Siebor, Lucie Amoureux, Catherine Neuwirth

**Affiliations:** ^1^Laboratoire de Bactériologie Médicale, Plateau Technique de Biologie, Centre Hospitalier Universitaire de Dijon, DijonFrance; ^2^Laboratoire Antibiotiques, Antifongiques: Physico-chimie, Synthèse et Activité Biologique, Faculté des Sciences de la Nature et de la Vie et Sciences de la Terre et de l’Univers, Université Abou Bekr Belkaid, TlemcenAlgérie; ^3^Département de Biologie, Faculté des Sciences de la Nature et de la Vie, Université Hassiba Benbouali, ChlefAlgérie; ^4^Département de Biologie, Faculté des Sciences de la Nature et de la Vie et Sciences de la Terre et de l’Univers, Université Abou Bekr Belkaid, TlemcenAlgérie; ^5^UMR 5569, Equipe “Pathogénes hydriques, Santé, Environnements" PHySE, Université de MontpellierFrance; ^6^INRA, UMR 1347 Agroécologie, DijonFrance

**Keywords:** *Escherichia coli*, extended-spectrum β-lactamase, 16S rRNA methylase, *armA*, *rmtB*, Algeria

## Abstract

The aim of this study, was to characterize the extended-spectrum-β-lactamases (ESBLs) producing clinical strains of *Escherichia coli* isolated between January 2009 and June 2012 from Algerian hospitals and to determine the prevalence of 16S rRNA methylase among them. Sixty-seven ESBL-producers were detected among the 239 isolates included: 52 CTX-M-15-producers, 5 CTX-M-3-producers, 5 CTX-M-1-producers, 2 CTX-M-14-producers, 2 SHV-12-producers and one TEM-167-producer. Among the ESBL–producing strains twelve harbored 16S rRNA methylase genes: 8 *rmtB* and 4 *armA*. *rmtB* was located on a IncFIA plasmid and *armA* was located either on a IncL/M or a IncFIA plasmid. RmtB-producing isolates were genotypically related and belonged to the sequence type ST 405 whereas ArmA-producing isolates belonged to ST10, ST 167, and ST 117. This first description of 16S rRNA methylases among *E. coli* in Algerian hospitals pointed out the necessity to establish control measures to avoid their dissemination.

## Introduction

The emergence of β-lactam resistance in *Escherichia coli* which is the most common cause of Gram-negative infections has become a significant health concern, mainly due to the production of extended-spectrum beta-lactamases (ESBLs) (Pitout and Laupland, 2008). Indeed, CTX-M ESBLs (more than 150 enzymes) have increased significantly in *Enterobacteriaceae*, particularly in *E. coli*, in most regions of the world (Cantón and Coque, 2006). Another worrisome feature is the emergence of isolates resistant to all clinically important aminoglycosides related to the production of 16S rRNA methylases. In 2003, the first 16S rRNA methylase gene, *armA*, was identified (Galimand et al., 2003). To date eleven 16S rRNA methylases genes (*rmtA*, *rmtB*, *rmtC*, *rmtD*, *rmtD2*, *rmtE*, *rmtF*, *rmtG*, *rmtH*, *armA*, and *npmA*) have been identified, *armA* and *rmtB* being the most frequently described in *Enterobacteriaceae* (Galimand et al., 2003; Bogaerts et al., 2007; Yamane et al., 2007; Fritsche et al., 2008). As these genes are usually located on plasmids they can easily transfer to other bacteria (Nagasawa et al., 2014).

In Algeria, the first CTX-M enzymes have been reported in 2005 in clinical strains of *Salmonella enterica* from Constantine, East of Algeria (Naas et al., 2005). Since then, several surveys in the north of Algeria have reported the presence of CTX-M-type enzyme and associated resistance to non-β-lactam markers in various species of *Enterobacteriaceae* (Baba Ahmed-Kazi Tani and Arlet, 2014). Concerning 16S rRNA methylases only ArmA has been detected to date. It was identified for the first time in *Enterobacteriaceae* strains isolated from an Algerian patient transferred to Belgium (Bogaerts et al., 2007). It was subsequently identified in clinical isolates of *S. enterica* in Constantine associated with CTX-M-3 or CTX-M-15 (Naas et al., 2009, 2011) and in clinical strains of *Salmonella* non-Typhi in Annaba associated with CTX-M-15 and CMY-2 (Bouzidi et al., 2011). Recently, it was also described in clinical isolates of *Klebsiella pneumoniae*, *Acinetobacter baumannii*, *Enterobacter cloacae*, and *Serratia marcescens* (Bakour et al., 2014; Belbel et al., 2014; Batah et al., 2015; Khennouchi et al., 2015).

In this study, we have characterized the ESBL-producing clinical strains of *E. coli* isolated between October 2008 and June 2012 in three hospitals in Western Algeria and determined the prevalence of 16S rRNA methylase genes among them.

## Materials and Methods

### Bacterial Strains

Two hundred and thirty-nine isolates of *E. coli* collected between October 2008 and June 2012 from three different hospitals located in North-Western Algeria (Tlemcen, Sidi Bel Abbes and Oran) were included. Two hundred and thirty-seven strains were isolated from patients admitted to the intensive care unit (ICU), surgery and neurosurgery wards. Two isolates have been recovered from hospital environment. Identification was performed using the API 20E system (BioMerieux) and/or matrix-assisted laser desorption/ionization time-of-flight mass spectrometry (MALDI-TOF MS).

### Antibiotic Susceptibility Testing

Twenty three antibiotics (Bio-Rad) were tested (**Table 1**). Antibiotic susceptibility testing was determined by disk diffusion method and the minimum inhibitory concentration (MICs) of colistin was determined by a dilution method in Mueller-Hinton (MH) broth (Bio-Rad) as described by the European Committee on Antimicrobial Susceptibility Testing^1^. Detection of ESBL production was performed by double disk synergy test (Jarlier et al., 1988).

**Table 1:** Antimicrobial resistance of extended-spectrum-β-lactamases producing *Escherichia coli* isolated from three hospitals in Algeria.

Antimicrobial agents	Resistance rate (%)			
	Tlemcen	Sidi Bel Abbes	Oran	Total
	(*n* = 35)	(*n* = 13)	(*n* = 19)	(*n* = 67)
Amoxicillin	100	100	100	100
Amoxicillin–clavulanic acid	82.8	92.3	42.1	73.1
Ticarcillin	100	100	100	100
Ticarcillin–clavulanic acid	97.1	100	89.5	95.5
Piperacillin	100	100	100	100
Piperacillin–tazobactam	40	15.4	0	23.9
Ertapenem	0	0	0	0
Imipenem	0	0	0	0
Cefoxitin	40	38.5	26.3	35.8
Cefotaxime	100	100	94.7	98.5
Ceftazidime	91.4	92.3	84.2	89.5
Cefepime	100	92.3	89.5	95.5
Aztreonam	100	100	100	100
Kanamicin	60	46.1	57.9	56.7
Gentamicin	77.1	76.9	63.2	73.1
Tobramycin	77.1	76.9	63.2	73.1
Amikacin	25.71	7.7	15.8	19.4
Netilmicin	48.6	61.5	57.9	53.7
Nalidixic acid	68.6	53.8	89.5	71.6
Ofloxacin	68.6	61.5	89.5	73.1
Ciprofloxacin	68.6	61.5	84.2	71.6
Colistin	0	0	0	0
Trimethoprim–Sulfamethoxazole	80	84.6	57.9	74.6

### Bacterial DNA Preparation, Gene Amplification, and Sequencing

Bacterial DNA preparation, gene amplification, sequencing, and nucleotide sequence analysis were performed as previously described (Siebor and Neuwirth, 2011). The primers used for the PCRs are listed in **Table 2**. All the isolates were analyzed for the presence of *bla*_CTX-M_, *bla*_TEM_, and *bla*_SHV_. The resistant strains to all aminoglycosides tested were further analyzed for the presence of *armA* and *rmtB*.

**Table 2:** Primers used for PCRs.

Amplified DNA	Primer	Oligonucleotide sequence (5′–3′)
	TEM–F_1_	ATAAAATTCTTGAAGACGAAA
*bla*_TEM_	TEM–F	CTTATTCCCTTTTTTGCGGC
	TEM–R	GGTCTGACAGTTACCAATGC
*bla*_SHV_	SHV–F	GGGTTATTCTTATTTGTCGC
	SHV–R	GTTAGCGTTGCCAGTGCTCG
*bla*_CTX-M_	CTX-M–F	ATGTGCAGYACCAGTAA
	CTX-M–R	ACCGCRATATCRTTGGT
*bla*_CTX-M_ group 1	CTX-M-gr1–F	TCGTCTCTTCCAGA
	CTX-M-gr1–R	CCGTTTCCGCTATTA
*bla*_CTX-M_ group 9	CTX-M-gr9–F	ATGGTGACAAAGAGAGTGCA
	CTX-M-gr9–R	ACAGCCCTTCGGCGATGATT
*armA*	*armA*-F	GTGCGAAAACAGTCGTAG
	*armA*-R	GAAGTCAGGATACACCAG
*rmtB*	*rmtB*-F	GAGACACCGATGAACATC
	*rmtB*-R	CCTTCTGATTGGCTTATC

### Genotyping of the Isolates Producing 16S rRNA Methylase

The strains were submitted to genotypic analysis by Pulsed-Field Gel Electrophoresis (PFGE) and Multilocus sequence typing (MLST). PFGE was performed following DNA digestion by *Xba*I as already described (Neuwirth et al., 1996). Restriction patterns were interpreted according to Tenover’s criteria (Tenover et al., 1995), and the isolates have been classified into different pulsotypes. Pulsotypes were compared by calculation of the Dice correlation coefficient with DendroUPGMA software^2^ and were clustered into a dendrogram by UPGMA. Strains were defined as having a clonal relationship at a criterium of 85% similarity (Ejrnaes et al., 2006). MLST was performed using seven conserved housekeeping genes of *E. coli* (*adk, fumC, gyrB, icd, mdh, purA*, and *recA*) as previously described (Wirth et al., 2006). The sequences types were determined by referring to the open-source software freely available at MLST Databases at the ERI, University College Cork website^3^.

### Mating Experiments and Plasmids Analysis

Mating experiments to attempt to transfer resistance genes were carried out using rifampicin resistant *E. coli* K-12 C600 recipient strain as described previously (Chen et al., 2007). Transconjugants were selected on Drigalski agar plates containing amikacin (4 mg/L), gentamicin (4 mg/L), and rifampicin (200 mg/L), on Drigalski agar plates containing cefotaxime (4 mg/L) and rifampicin (200 mg/L), on Drigalski agar plates containing gentamicin (4 mg/L) and rifampicin (200 mg/L) and on Drigalski agar plates containing amikacin (30 mg/L) and rifampicin (200 mg/L).

Identification of plasmids by PCR-based replicon typing was performed on the donor strains and their transconjugants as described previously (Carattoli et al., 2005). Plasmid sizes were determined by digestion of whole-genome DNA with S1 endonuclease to linearize the plasmids and PFGE to separate them. Plasmids obtained were transferred to nylon membranes and hybridized with digoxigenin-labeled specific probes [*bla*_CTX-M_ group 1, *bla*_TEM_, *armA*, *rmtB*, and replicon groups (IncL/M, IncFIA, IncFIB, and IncN)] (Barton et al., 1995).

## Results

### ESBL Producers

Sixty-seven ESBL-producing isolates have been detected: 35 from Tlemcen hospital, 13 from Sidi Bel Abbes hospital and 19 from Oran hospital. Isolates were mainly recovered from ICU (58.2%), followed by 26.9% from surgery department. Eighteen strains (26.9%) were recovered from wound pus, 17 (25.4%) from tracheal aspirate, 17 (25.4%) from urine and urinary catheters, 7 (10.4%) from rectal swabs, 4 (6%) from bedsores, 1 (1.5%) from catheter, 1 (1.5%) from gastric tube, and 2 (3%) from hospital environment. Among these strains 64 isolates (95.5%) carried *bla*_CTX-M_ (52 *bla*_CTX-M-15_, 5 *bla*_CTX-M-3_, 5 *bla*_CTX-M-1_, 2 *bla*_CTX-M-14_), while 2 carried *bla*_SHV-12_ and 1 *bla*_TEM-167_.

All the ESBL-producing isolates remained susceptible to imipenem, ertapenem, and colistin and most of them to piperacillin-tazobactam (76%), cefoxitin (64.2%), and amikacin (80.6%). However most of the strains were resistant to all other antibiotics tested (>53%) (**Table 1**).

### Characterization of 16S rRNA Methylase-Producers (**Table 3**; Supplementary Figure S1)

Twelve isolates were resistant to all aminoglycosides tested of which four strains harbored *armA* and 8 *rmtB*. *ArmA* was detected among CTX-M-15, CTX-M-1, and TEM-167-producers whereas *rmtB* was detected only among CTX-M-15-producers (**Table 3**). The eight RmtB-producing strains all originated from Tlemcen hospital belonged to pulsotype A and to ST 405. A wider diversity was observed among the four ArmA-producers that belonged to three different pulsotypes (B, C, and D) and three different ST: ST10 (one isolate from Sidi Bel Abbes hospital), ST167 (one from Tlemcen hospital), and ST117 (two isolates from Oran hospital).

**Table 3:** Characteristics of the 16S rRNA methylase producing *E. coli* strains isolated in Tlemcen, Sidi Bel Abbes, and Oran hospitals, Algeria.

Strain	Hospital	Ward	Isolation date	Sample	ESBL (plasmid)	16S rRNA methylase (plasmid)	PFGE pulsotypes	MLST
T E62	Tlemcen	Neurosurgery	19/10/2008	Bedsore	*bla*_CTX-M-15_[Inc L/M 55 kb (T+)^3^]	*armA* [same Inc L/M 55 kb (T+)]	C	ST 167 (CC ST 10)
S E23	Sidi Bel Abbes	Neurosurgery	18/03/2010	Pus	*bla*_CTX-M-15_[Inc L/M 55 kb (T+)]	*armA* [same Inc L/M 55 kb (T+)]	D	ST 10 (CC ST 10)
0 E11	Oran	ICU**^1^**	16/12/2010	Infected wound	*bla*_TEM-167_[Inc N 40 kb (T+)]	*armA*[Inc FIA size nd^5^ (T+)]	B	ST 117
0 E13	Oran	Neurosurgery	16/12/2010	Gastric tube	*bla*_CTXM-1_[Inc N 40 kb (T+)]	*armA*[Inc FIA size nd (T-)]	B	ST 117
T E114	Tlemcen	ICU	01/04/2010	Tracheal aspirate	*bla*_CTX-M-15_[Inc FIA About 100 kb (T-)^4^]	*rmtB* [same Inc FIA About 100 kb (T-)]	A	ST 405
T E116	Tlemcen	ICU	02/05/2010	Rectal swab	*bla*_CTX-M-15_[Inc I1 About 100 kb (T+)]	*rmtB* [Inc FIA About 85 kb(T+)]	A	ST 405
T E117	Tlemcen	ICU	20/05/2010	Tracheal aspirate	*bla*_CTX-M-15_[Inc I1 About 100 kb (T+)]	*rmtB* [Inc FIA About 85 kb (T+)]	A	ST 405
T E118	Tlemcen	ICU	01/06/2010	Tracheal aspirate	*bla*_CTX-M-15_[(Inc I1 About 100 kb (T+)]	*rmtB* [Inc FIA About 85 kb (T+)]	A	ST 405
T E120	Tlemcen	ICU	06/01/2011	Tracheal aspirate	*bla*_CTX-M-15_[Inc I1 About 100 kb (T+)]	*rmtB* [Inc FIA About 85 kb (T+)]	A	ST 405
T E124	Tlemcen	ICU	27/02/2011	Rectal swab	*bla*_CTX-M-15_[Inc FIA About 100 kb (T-)]	*rmtB* [same Inc FIA About 100 kb (T-)]	A	ST 405
T E133	Tlemcen	ENT^2^	21/04/2011	Catheter	*bla*_CTX-M-15_[Inc FIA About 100 kb (T-)]	*rmtB* [same Inc FIA About 100 kb (T-)]	A	ST 405
T E134	Tlemcen	ICU	26/04/2011	Urine	*bla*_CTX-M-15_[Inc FIA About 100 kb (T-)]	*rmtB* [same Inc FIA About 100 kb (T-)]	A	ST 405

*^1^ICU, intensif care unit. ^2^ENT, ear nose throat surgery. ^3^T+, obtention of transconjugant. ^4^T-, no transconjugant. ^5^nd, non determined.*

In the eight isolates, *rmtB* was located on a IncFIA plasmid. In four cases *bla*_CTX-M-15_ was located on the same plasmid while it was located on a IncI1 plasmid in the other four cases. IncFIA was transferred only in the presence of IncI1. *armA* was located either on a transferable IncL/M plasmid (with *bla*_CTX-M-15_) or on a IncFIA plasmid (in these two cases *bla*_CTX-M-1_ or *bla*_TEM-167_ were located on a IncN plasmid).

## Discussion

Our results are in accord with previous studies and confirm that CTX-M-15 is the predominant ESBL encountered among *E. coli* in Algerian hospitals (Ramdani-Bouguessa et al., 2006; Baba Ahmed-Kazi Tani et al., 2013). The same situation is also observed in Tunisia (Mamlouk et al., 2006) and Morocco (Girlich et al., 2014) illustrating the large spread of CTX-M-15-producing *E. coli* in the north African countries. Nevertheless, the CTX-M-1, CTX-M-14, SHV-12, and TEM-167 ESBLs also detected in our study had never been reported to date among *E. coli* strains in Algeria. There is a single description of CTX-M-14-producing *S. enterica* collected from Draa-El-Mizan hospital, east of Algeria (Iabadene et al., 2009) whereas SHV-12 has already been detected in clinical isolates of *K. pneumoniae* and *E. cloacae* in Algeria (Baba Ahmed-Kazi Tani and Arlet, 2014). There is no description of TEM-167 to date, only *bla*_TEM-167_ is available in GenBank (FJ360884).

Our study further highlights the emergence of 16S rRNA methylase enzymes among ESBL-producing *E. coli* strains in the west of Algeria. Among our collection, the prevalence rate of 16S rRNA methylase determinants was 18%, which is very high compared to those reported in Japan (0.03%) (Yamane et al., 2007), in Turkey (0.7%) (Bercot et al., 2010), in France (1.3%) (Bercot et al., 2008), and in China (10%) (Wu et al., 2009). The detection of RmtB-producing *E. coli* is an interesting finding of our study. Indeed the RmtB enzyme has never been reported in Algeria. The eight isolates producing RmtB were genotypically related and belonged to ST405. Interestingly ST405 associated or not with production of CTX-M-15 ESBL is increasingly reported worldwide and belongs to the high-risk clones in the dissemination of antibiotic resistance (Woodford et al., 2011). The association of *bla*_CTX-M-15_ and *rmtB* on a conjugative plasmid (also carrying the plasmid-mediated fluoroquinolone eﬄux pump gene *qepA*) in one ST405 *E. coli* isolate has been reported in the United States (Tian et al., 2011) but the plasmid was not typed by PCR-based replicon.

All RmtB-producers have been recovered in Tlemcen Hospital and seven of them were collected in ICU suggesting patient cross contamination in this ward.

Therefore there is a need to reinforce the compliance of health-care workers with the infection control measures. The epidemiological results concerning ArmA-producers is quite different: the four isolates recovered in three hospitals in Western Algeria belonged to three different pulsotypes suggesting horizontal spread of the resistance determinants among *E. coli*. There are several reports of ArmA-producers among different species of *Enterobacteriaceae* and also *A. baumannii* from Algerian Hospitals (Bogaerts et al., 2007; Naas et al., 2009, 2011; Bouzidi et al., 2011; Bakour et al., 2014; Belbel et al., 2014; Batah et al., 2015; Khennouchi et al., 2015). The production of 16S rRNA methylase together with the production of ESBL observed in our study (RmtB plus CTX-M-15 or ArmA plus CTX-M-15) corresponds to an emerging and threatening combination reported worldwide (Bogaerts et al., 2007; Khennouchi et al., 2015). To our best knowledge two combinations detected in our study were not reported before: ArmA plus CTX-M-1 and ArmA plus TEM-167 in two isolates clonally related (OE11 and OE13) recovered simultaneously from Oran’s hospital.

Our data indicated that the diversity of ESBL among *E. coli* is growing in Algerian hospitals (CTX-M-1, CTX-M-14, CTX-M-15, TEM-167, and SHV-12) and that new mechanisms are emerging (16S rRNA methylases). In our study 11 out of 12 isolates that co-produced ESBL and methylase were resistant to all antibiotics tested except carbapenems and colistin, making of them the only available therapeutic option for these strains. Therefore the public health impact is potentially important.

## Conclusion

There is a need for implementation of antimicrobial stewardship programs in Algerian hospitals to make the best use of the available antibiotics and to avoid as much as possible the unreasonable use of broad-spectrum molecules leading to the emergence of resistant isolates. Also strict control measures have to be elaborated to avoid the spread of multiresistant organisms such as those described here that co-produced ESBL and 16S rRNA methylase.

## Author Contributions

AA: Selected the isolates, performed the characterization of the ESBL, of the methylase, the analysis by PFGE, the MLST analysis and manuscript preparation. MD: Designed the study. CdC and LA: Participated to genotyping and interpretation. ES: Sequence analysis and manuscript preparation. AH and SS: Performed plasmid analysis. CD: Performed PFGE analysis (dendrogram). CN: Choice of the isolates, choice of the tools, interpretation of the results and manuscript preparation.

## Conflict of Interest Statement

The authors declare that the research was conducted in the absence of any commercial or financial relationships that could be construed as a potential conflict of interest.

## Footnotes

1 www.eucast.org

2 http://genomes.urv.cat/UPGMA/

3 http://mlst.ucc.ie/mlst/dbs/Ecoli
