# Supplementary material for: Occurence of ArmA and RmtB Aminoglycoside Resistance 16S rRNA Methylases in Extended-Spectrum β-Lactamases Producing Escherichia coli in Algerian Hospitals
Source: Front Microbiol. 2016 Sep 12;7:1409. doi: 10.3389/fmicb.2016.01409 (PMC5018485; doi:10.3389/fmicb.2016.01409)

UPGMA cluster analysis of XbaI-generated pulsotypes constructed with Dice coefficient. Isolate designation is indicated on the dendrogram branches. The Dice's coefficient scale is at the bottom of the dendrogram.

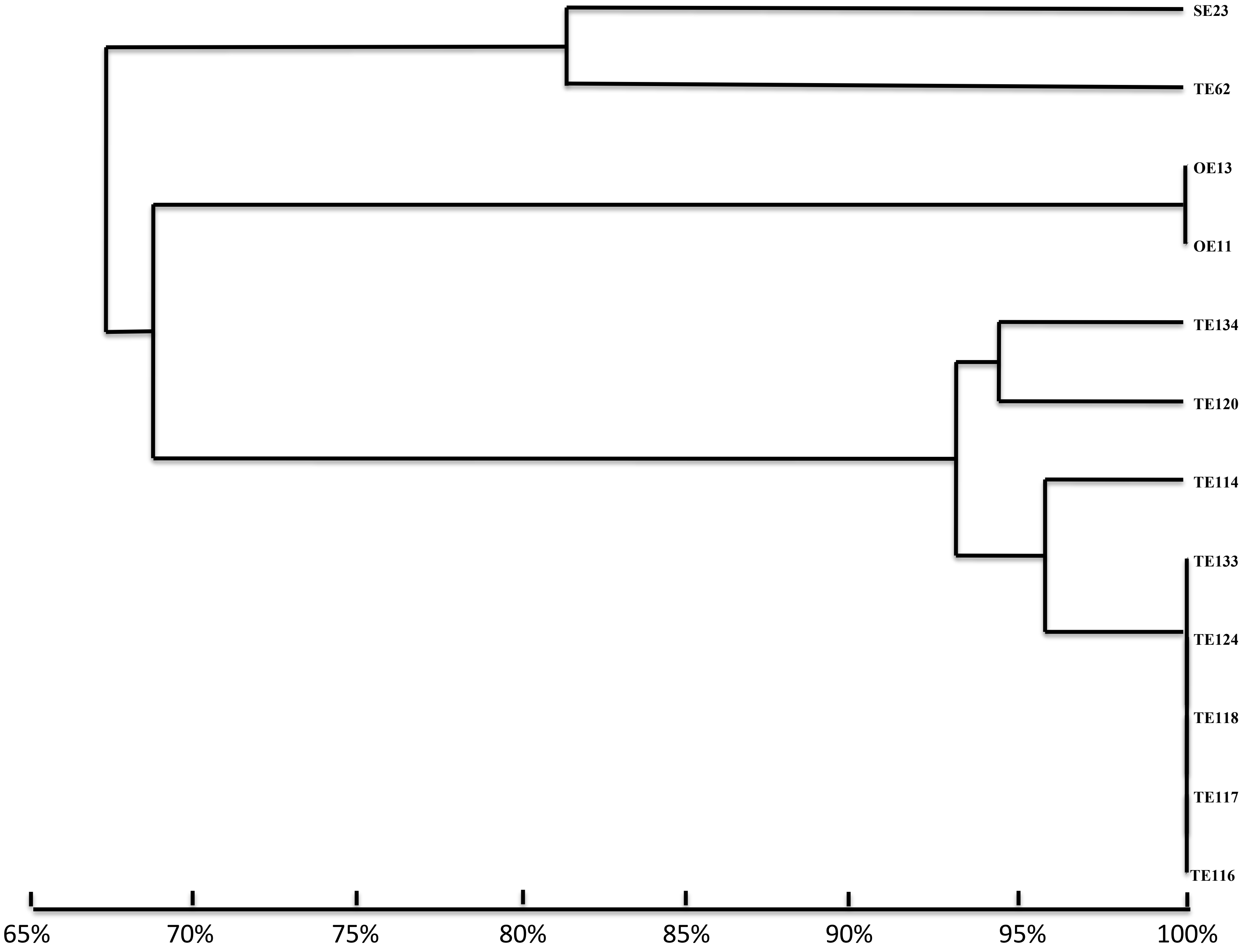

Supplement: Supplementary file 1 [file Image_1.PDF]
